# Clinical Characteristics and Outcomes in Patients with Chronic HBV Infection and Hospitalized for COVID-19 Pneumonia: A Retrospective Cohort Study

**DOI:** 10.3390/v17010040

**Published:** 2024-12-30

**Authors:** Lucio Boglione, Maria Grazia Crobu, Mario Pirisi, Carlo Smirne

**Affiliations:** 1 Department of Translational Medicine, Università del Piemonte Orientale, 28100 Novara, Italy; lucio.boglione@uniupo.it (L.B.); mario.pirisi@med.uniupo.it (M.P.); 2 Laboratory of Molecular Virology, Maggiore della Carità Hospital, 28100 Novara, Italy; mcrobu@cittadellasalute.to.it; 3 Clinical Biochemistry Laboratory, City of Health and Science University Hospital, 10126 Turin, Italy; 4 Internal Medicine Unit, Maggiore della Carità Hospital, 28100 Novara, Italy

**Keywords:** COVID-19, SARS-CoV-2, hepatitis B virus, chronic hepatitis B, HBV reactivation, coinfection, superinfection, liver injury, prognosis, liver function

## Abstract

The effects of a concomitant infection of hepatitis B virus (HBV) and severe acute respiratory syndrome coronavirus 2 (SARS-CoV-2) are still debated, with a recognized major risk of HBV reactivation during immune-suppressive treatments. The aim of this study was to determine the prevalence and predictive factors of HBV reactivation in a cohort of hospitalized patients with coronavirus disease 2019 (COVID-19) and a current or past hepatitis B infection. In a monocentric retrospective observational study, we enrolled all consecutive hospital admitted patients with COVID-19 pneumonia and a positive HBV serology (N = 84) in our Infectious Diseases Unit from April 2021 to December 2023. We identified 18 (21%) HBsAg-positive/anti-HBc-positive, 41 (49%) HBsAg-negative/anti-HBc-positive/anti-HBs-positive, and 25 (30%) HBsAg-negative/anti-HBc-positive/anti-HBs-negative subjects. The overall rate of hepatitis flare was 10.7%, without any HBsAg seroreversion, severe HBV reactivation, and/or need for new HBV antiviral therapy introduction. Systemic corticosteroid treatment for COVID-19 and baseline anti-HBsAg status were associated with this risk of HBV reactivation. In conclusion, the overall risk of hepatitis flares in hospitalized COVID-19 was reasonably low, with higher doses of corticosteroids treatment being the major risk factor for HBV reactivation, and anti-HBs-positive serological status as a protective element.

## 1. Introduction

Severe acute respiratory syndrome coronavirus 2 (SARS-CoV-2) is the cause of the coronavirus disease 2019 (COVID-19), a global pandemic having important consequences for public health, world economy, and clinical aspects [1]. The main clinical manifestation is acute respiratory syndrome with lung involvement and interstitial pneumonia [2], although multiorgan illness due to cardiovascular, neurological, and gastrointestinal failure has been widely described [3]. Gastrointestinal symptoms are more often associated with the outset of viral infection, especially in outpatients, and are generally related to a mild-to-moderate course of the disease [4]. It is well known that liver function abnormalities are frequently observed during the acute phase of viral illness; however, this aspect may be related to both direct viral action on hepatocytes and systemic inflammation due to the “cytokine storm”, as previously and thoroughly documented [5,6]. The prevalence and size of the liver injury during COVID-19 can depend on several factors, such as local epidemiology, severity of illness, grade of hypoxemia, presence of comorbidities, but also use of drugs with potential hepatotoxicity [7]. Due to these important variables, it is reasonable to state that the overall mortality rate may occasionally be influenced by hepatic involvement, even in the absence of an underlying liver disease, although this effect should be considered as part of a larger whole in which some of the factors mentioned previously—such as the severity of COVID-19 illness, comorbidities, and the presence of acute respiratory distress syndrome (ARDS) or multiorgan failure—still play a prevalent role [8,9].

Hepatitis B virus (HBV) is an important cause of chronic hepatic infection related to liver disease, cirrhosis, and hepatocellular carcinoma, affecting more than 250 million of people worldwide [10]. Despite the quite relevant number of patients with a concomitant infection of SARS-CoV-2 and HBV, the actual clinical impact of this viral interaction is still unclear. The overall prevalence of HBV infection in SARS-CoV-2-positive subjects ranges from 0.8% to 14.0%, depending on the specific features of the studied populations, such as geographical origin, availability of complete HBV diagnosis, sample size, and local epidemiology [11]. Furthermore, the clinical impact of HBV infection on COVID-19 outcomes is another point of discussion: some studies evidenced an increased risk of disease severity and mortality, while others observed no significant effects [12,13]. Finally, few data are currently available about the role of SARS-CoV-2 infection on the clinical and virological course of HBV, especially given the heterogeneous stages of HBV hepatitis in the various study groups [6,14]. Regardless, the risk of HBV reactivation (both virological and biochemical) has been well documented with most of the immunosuppressive therapies [15] and in the course of COVID-19 [16,17], but the overall rate of severe liver injury has been reasonably low [18].

Based on these premises, the aim of this study was to document the risk of HBV reactivation in a cohort of hospitalized patients affected by COVID-19 in Italy.

## 2. Materials and Methods

### 2.1. Study Design

This was a retrospective study including all patients ≥18 years affected by SARS-CoV-2 and chronic current or past HBV infection, admitted from April 2021 to December 2023, at our center of Infectious Diseases at Sant’Andrea Hospital, Vercelli (Italy), for COVID-19 pneumonia. All subjects gave written informed consent to their participation in this research, which was conducted in strict adherence to the principles of the Declaration of Helsinki of 1975, as revised in 2000. The study protocol was approved by the local institutional ethics committee: Comitato Etico Interaziendale ASL VC, https://aslvc.piemonte.it/organizzazione/responsabili-delle-strutture/organismi-collegiali/comitato-etico, IRB code 0026301 (accessed on 20 October 2024).

### 2.2. Data Collection

Patients positive for hepatitis C (HCV)-RNA or markers of human immunodeficiency virus (HIV) (i.e., anti-HIV antibodies ± HIV-RNA), hepatitis D virus (HDV), or hepatitis E virus (HEV) were excluded from this study. SARS-CoV-2 infection was defined as the occurrence of a positive specific polymerase chain reaction (PCR) test, in the presence or absence of symptoms at hospital admission. The selected patients had also complete HBV serology and liver function tests available with at least alanine transaminase (ALT) levels. The baseline studied characteristics included: demographic data, functional and clinical status, biochemical analyses, clinical and therapeutical course. Hepatitis B surface anti-gen (HBsAg) was used to determine the current status of HBV infection (i.e., active or past); hepatitis B core antibody (anti-HBc) was tested in all subjects with HBsAg-negative status with or without the presence of hepatitis B surface antibody (anti-HBs). HBV reactivation during hospitalization due to COVID-19 was defined as at least one of these different outcomes: HBV-DNA rise from the baseline level or reappearance of HBV-DNA in individuals without detectable viral DNA; loss of anti-HBs with HBsAg reappearance (the so-called reverse HBsAg seroconversion); hepatitis flare with increase in ALT levels with or without symptoms when the main extrahepatic causes of increased transaminases had been carefully excluded [18].

The following routine assays were used in our laboratory: for HBsAg detection, LIAISON^®^ XL MUREX HBsAg Quant (DiaSorin, Saluggia, Italy); for anti-HBc detection, LIAISON^®^ Anti-HBc and LIAISON^®^ HBc IgM (DiaSorin); for anti-HBs detection, LIAISON^®^ XL MUREX Anti-HBs & Plus (DiaSorin); for anti-HCV detection, LIAISON^®^ XL MUREX HCV Ab (DiaSorin); for anti-HDV detection, HDV Ab—ELISA (Dia.pro Diagnostic Bioprobes, Sesto San Giovanni, Italy); for anti-HEV detection, LIAISON^®^ Murex Anti-HEV IgG and LIAISON^®^ Murex Anti-HEV IgM (DiaSorin); for anti-HIV detection, LIAISON^®^ XL MUREX HIV Ab/Ag (DiaSorin); for HBV-DNA detection, Alinity m HBV (Abbott Laboratories, Abbott Park, IL, USA); for HCV-RNA detection, Alinity m HCV (Abbott Laboratories); for SARS-CoV-2 RNA detection, ALINITY m Resp-4-Plex AMP KIT (Abbott Laboratories).

### 2.3. Statistical Analysis

Continuous variables are summarized as medians and interquartile ranges (IQRs). Categorical variables are described as frequencies and percentages. All data were assessed for normality using a Shapiro–Wilk test, and categorical data were compared using a Mann–Whitney or Kruskal–Wallis statistical test, as appropriate. To investigate continuous data, the Spearman rank correlation was utilized. Associations were calculated using the χ^2^-test. Multivariate logistic regression analysis with stepwise forward selection was performed to evaluate the factors related to HBV reactivation, with *p* values less than 0.05 as the criteria for model inclusion. All *p*-values are two-tailed and are considered statistically significant when <0.05. Statistical analyses were conducted by using SPSS software package ver. 26.0 (IBM, Chicago, IL, USA).

## 3. Results

### 3.1. Baseline Characteristics of the Patients

The baseline characteristics of patients are reported in Table 1. The overall number of native liver subjects admitted with COVID-19 and available HBV serology was 386. Three hundred and two individuals (78.2% of the screened subjects) had no previous exposure to HBV infection and, consequently, were excluded from this analysis. The remaining 84 (21.8% of the screened population) were Caucasian anti-HBc-positive patients, further divided into HBsAg-positive (N = 18, 21.4%); HBsAg-negative/anti-HBs-positive (N = 41, 48.8%); HBsAg-negative/anti-HBs-negative (N = 25, 29.8%). Notably, concerning comorbidities, no patient had an active rheumatological disease with the need for concomitant immunosuppressive or systemic corticosteroid treatments. The presence of circulating HBV-DNA was detected in four HBsAg-positive patients (22.2% of this subcategory) and in one anti-HBc-positive/anti-HBs-negative subject (4% of this subcategory). Three HBsAg-positive individuals (16.7% of this subcategory) were receiving antiviral therapy with nucleoside analogue (NA) entecavir before hospital admission. No patient had established cirrhosis.

### 3.2. Outcomes in the Study Population

Table 2 summarizes the clinical outcomes in the study population according to HBV status: time of hospitalization, intensive care unit (ICU) admission, continuous positive airway pressure (CPAP)/noninvasive ventilation (NIV) support, sepsis, and/or death within one month of hospital admission.

HBV reactivation leading to an increase in HBV-DNA was observed in two patients with HBsAg-positive status (11%, none in previous treatment with NAs) and in two with an anti-HBc-positive/anti-HBs-negative pattern (8%), of which one was negative at baseline. However, the rise in HBV-DNA was, in all cases, below 1.0 Log10 from baseline, and no antiviral therapy was needed. HBsAg seroreversion was not detected.

Hepatitis flare with an increase in ALT levels was observed in 10.7% of the studied population (again, not in patients being treated with NAs) and occurred more frequently in HBsAg-positive/anti-HBc-positive (16.7%) and HBsAg-negative/anti-HBc-positive/anti-HBs-negative (16.0%) patients than in HBsAg-negative/anti-HBc-positive/anti-HBs-positive subjects (*p* = 0.003). In almost all cases, the rise was between 2.5 and 10× the upper normal level (ULN) of the local laboratory, defined as 65 U/L.

In all four patients with viremia increase, HBV-DNA returned to levels that were comparable to those at baseline at a three months follow-up; within the same time interval, a similar trend was observed in the nine subjects who experienced a hepatitis flare. When taking death events into account, in all cases, they were attributed by the treating physicians to extrahepatic causes and not directly to HBV liver failure, although it is worth noting that those who were anti-HBs-positive had lower death rates than the other two subgroups.

### 3.3. Factors Associated with HBV Reactivation

The considered factors possibly associated with HBV reactivation included age, sex, comorbidities, ICU admission, CPAP/NIV support, sepsis, presence of circulating HBV-DNA, anti-HBs positivity, and COVID-19 treatment with corticosteroids and/or remdesivir. Multivariate analysis—performed among those variables with *p* < 0.05 at univariate analysis—identified the following as possible independent predictors of HBV reactivation: ICU admission (OR 3.914, *p* = 0.008) and corticosteroid treatment (OR 4.367, *p* = 0.002). Conversely, anti-HBsAg-positive status was found to be a protective factor against viral reactivation (OR 0.804, *p* = 0.014). These analyses are extensively reported in Table 3.

## 4. Discussion

Data regarding viral superinfections/coinfections in patients with COVID-19 are limited and still emerging. According to the Centers for Disease Control and Prevention (CDC), a superinfection is an infection following a previous infection, while a coinfection is an infection concomitant with the initial infection. Although the two terms are used interchangeably, they are different clinical entities, which is particularly relevant when discussing SARS-CoV-2 infection. In any case, superinfections and coinfections can potentiate microbial pathogenesis, increasing COVID-19 morbidity and mortality [19]. Regarding superinfections, they have been demonstrated to trigger latent virus reactivations through different types of stimuli, including immunosuppression [20]. Not surprisingly, patients with severe COVID-19 are characterized by impaired immunity, hyperinflammation, lymphopenia, and cytokine storm [21]. The viruses so far more frequently reported in coinfection with SARS-CoV-2 are influenza A virus and Epstein–Barr virus (EBV), while EBV, herpes simplex virus 1, and cytomegalovirus have been more frequently observed in superinfection/reactivation cases [22]. In rather few instances, hepatitis B virus (HBV) reactivation in chronic patients has also been documented [23,24]. In both reactivation and coinfection groups, patients frequently report cardiovascular disease, diabetes, and immunosuppression as comorbidities, acute kidney injury as a complication, and lymphopenia and elevated D-dimer/C-reactive protein levels [25,26,27,28].

Focusing on SARS-CoV-2 and HBV interactions, the latter is a recognized major public health problem worldwide due to its causal role in acute and chronic liver damage [29]. Similarly, there is growing evidence that COVID-19 can cause abnormal liver function and hepatic impairment [30,31]. However, when analyzing the direct effects of COVID-19 on chronic hepatitis B (CHB), most evidence supports that SARS-CoV-2 infection may not increase the risk of severe liver injury, provided that baseline liver function is preserved. Conversely, there are also many reports on the possible effects of CHB on SARS-CoV-2 infection (including its direct complications, mortality, and incidence of severe forms). With regard to this latter issue, it can be assumed that the immune status of the host is influenced to some extent by CHB, which in turn may affect the consequences of infection with SARS-CoV-2 [32].

Considering both diseases at the same time and their mutual interactions, most studies have so far produced inconsistent results on whether SARS-CoV-2 and HBV co- and superinfections increase the overall risk of death and critical illness [33,34]. Furthermore, whether there is a difference in the risk of such unfavorable outcomes between the different CHB infection phases and stages of liver damage is still a matter of debate. As a matter of fact, there are some reports describing how patients with SARS-CoV-2/HBV have an increased global risk of poor prognosis (such as ICU admission and/or death), which is—at least to some extent—mediated by abnormal liver function [35], but this finding has not been confirmed by others [36]. What emerged from our study population was that both the overall ICU admission and the mortality rates were reasonably low (14.3% and 4.8%, respectively) without significant differences between the different HBV serological categories. These rates are slightly lower than those reported for mono-infected SARS-CoV-2 subjects for the corresponding pandemic waves [37,38]. One possible simple explanation for this could be the fact that Italy, i.e., the population in which this study was conducted and which was so hard hit by the first waves of the disease, achieved one of the highest SARS-CoV-2 vaccination rates in the world: by the end of September 2023, 90.25% of the population aged ≥12 years completed the first immunization schedule [39]. Precisely, SARS-CoV-2 vaccination is a recognized protective factor against the most clinically severe forms of the disease [40].

Coming back to the previously cited and not fully answered issue—i.e., if patients with SARS-CoV-2 and HBV co-superinfection are at risk of greater direct or indirect liver injury—it should be considered that this interesting topic is rather complex and multifaceted. Specifically, the natural course of this co-superinfection can lead to several grades of hepatic involvement, depending on multiple extrahepatic factors, such as patient status, illness severity, comorbidities, and/or immunosuppressive therapies (including SARS-CoV-2 antiviral treatments) [22,36,41,42,43,44]. What is well established is that, in most cases, the advanced stage of COVID-19 is characterized by a hyperinflammatory state—the previously cited cytokine storm—which can result in multiorgan impairment, including of the liver itself. However, particularly during the first wave of pandemic, many confounding factors were related to liver impairment despite having no direct causal relation to HBV status. As a consequence, the reported available data on these two viral co-superinfections are not fully conclusive, at least for this period [22]. In more detail, the presence of ARDS requiring invasive or noninvasive (CPAP/NIV) mechanical ventilation approaches, and generally speaking the occurrence of ICU admission, can increase the risk of liver impairment [11]. In other words, for the same baseline stage of liver damage, it is the overall COVID-19 severity that seems to be the most important factor leading to hepatic involvement, in addition to more broadly affecting the overall prognosis [8], as more generally demonstrated for SARS-CoV-2 infections not concurrent with HBV [45,46].

All these considerations aside, the data currently available in the literature show that HBV co-superinfection can at most slightly affect clinical outcomes and liver function [14,47,48,49,50,51,52]. These negative reports were confirmed in our research. As a matter of fact, in our study population, all death events were primarily attributed by the treating physicians to extrahepatic causes and none—directly or indirectly—to the underlying CHB. This result could be partially explained by the fact that none of the patients in our case series had advanced liver fibrosis or established cirrhosis. In this respect, the patients with the most advanced liver disease are those who have the worst prognosis during SARS-CoV-2 intercurrent infections, as has been well documented for cirrhosis, including the HBV-derived forms [53,54,55,56,57]. However, an additional consideration is that this evidence was mainly generated during the first and the second waves of the pandemic, while more recent studies centered on patients recruited in the same time frame as the present study did not always confirm these findings [36,44]. Finally, we can speculate that our study population, though not randomized, was well balanced amongst the three considered HBV serological patterns concerning both the key demographic characteristics and the prevalence of comorbid diseases, which allowed us to analyze both the overall and the liver-related prognoses without these albeit important parameters as possible confounding factors, with a methodology similar to that reported by other groups [36,44].

An additional important issue when addressing liver damage is the potential for HBV reactivation. This has been generally recognized as a complication of immunosuppressive treatments, particularly cytotoxic chemotherapy, in individuals with prior exposure to HBV, as well as those who are chronically infected with hepatitis B. In any case, the key initial event is a loss of HBV immune control, which leads to an increase in HBV-DNA, with also possible hepatitis flares [58,59]. From this point of view, antiviral therapy for SARS-CoV-2 can also act as a major trigger. The most common current therapies for COVID-19 involved in the HBV reactivation are tocilizumab, baricitinib, and, not surprisingly, systemic corticosteroids [16,17]. In this respect, the highest risk of reactivation is for those who are HBsAg-positive and receiving tocilizumab [60], as previously well documented in subjects not affected by SARS-CoV-2 [61,62]. However, in our study population, no individual received such treatment, so this specific issue could not be addressed.

Instead, our research confirmed that moderate to high doses of corticosteroids (≥10 mg of methylprednisolone or equivalent dosages) are possibly related to hepatitis flares [17]. According to our local diagnostic and therapeutic care pathway, high doses of dexamethasone (6 mg/daily for 5–10 days) were administered in a large part (58.3%) of our cohort. The rationale for this approach was derived from the RECOVERY (Randomised Evaluation of COVID-19 Therapy) trial that compared patients treated with dexamethasone with subjects receiving usual care; the reported incidence of death was significantly lower in the dexamethasone group among individuals receiving invasive mechanical ventilation (29.3% vs. 41.4%) or oxygen without invasive mechanical ventilation (23.3% vs. 26.2%) [63]. Based on this premise, dexamethasone therapy was recommended by both the Committee for Medicinal Products for Human Use (CHMP) of the European Medicines Agency and the Italian Medicines Agency as soon as September 2020 in subjects with respiratory failure. The administration of this therapeutical regimen, especially if early, was then confirmed as being able to significantly improve the prognosis of people with COVID-19 pneumonia in most of the studies conducted until now [64,65]. However, while this strategy is generally well tolerated, in subjects with present and even past HBV infection, it can result in an increased risk of HBV reactivation, even if administered just for a few days [66]. Hence, routine testing for HBV serology is recommended in all individuals prior to commencing these immunosuppressive treatments [67]. In our experience, corticosteroid treatment—justified by the fact that the vast majority of our patients required oxygen supplementation—was confirmed as the major independent predictive factor of hepatitis flares, although the most severe event (i.e., reverse HBsAg seroconversion) was not observed.

One additional consideration is that—probably due to the limited duration of the administered corticosteroid therapy according to the protocol—hepatitis flares occurred quite frequently (10.7%), but no subject required HBV antiviral therapy and/or other specific supportive treatments, and the transaminase levels normalized after the COVID-19 resolution. Conversely, anti-HBs status was found to be a strong protective factor against HBV reactivation, suggesting that this test may be useful for prospectively estimating the risk of hepatitis flares in patients with COVID-19. By contrast, as expected, those most susceptible to liver enzyme elevation were HBsAg-positive.

In any case, when interpreting the results, some major limitations of this study should be kept in mind, such as its retrospective design, the relatively limited sample size, the different characteristics of the patients included during various waves of the pandemic, the use of local protocols for COVID-19 treatment (including corticosteroids), and—most important of all—the inclusion of only hospitalized patients with frequent systemic illness and/or multiorgan involvement. Aside from these considerations, these latter aspects, to the best of our knowledge, are not formally reported in the current COVID-19 guidelines, and have been scarcely discussed in the literature [22,24,35,68]. Thus, it is our opinion that they should be prospectively confirmed in larger studies.

The only other variable that our research found to be associated with HBV reactivation at multivariate analysis, i.e., ICU admission, was likely attributable to the higher and/or more prolonged dose of corticosteroids that was administered before and during the stay in the critical care facility [11,67,69,70], as suggested by other authors, although there are few reports documenting this possibility in patients with the most severe forms of COVID-19 and not receiving any corticosteroid treatment [23].

## 5. Conclusions

HBV reactivation in a cohort of Italian patients hospitalized for COVID-19 was observed in 10.7%, but no seroreversion or clinically relevant hepatic flare events were documented. Corticosteroids, particularly at higher doses, were predictive of HBV reactivation, while anti-HBs-positive status was a protective factor, suggesting that it may be detected at baseline for a better risk stratification.

## Figures and Tables

**Table 1:** Baseline characteristics of included patients (N = 84). Percentages refer to each column in this table, unless otherwise indicated. Bold values denote statistical significance at *p* < 0.05 level.

	HBsAg-Positive/ Anti-HBc-Positive	HBsAg-Negative/Anti-HBc-Positive		*p*
		Anti-HBs-Positive	Anti-HBs-Negative	
Patients (N)	18 (21.4) ^1^	41 (48.8) ^1^	25 (29.8) ^1^	NS
Age (years)	61 [58.5–72.5]	74 [69–81]	73 [65–84]	**<0.001**
Male sex (N)	15 (83.3)	30 (73.2)	18 (72.0)	NS
Liver stiffness (kPa) ^2^	7.5 [6.2–9.5]	7.8 [6.1–9.4]	7.1 [5.4–8.9]	NS
Comorbidities (N)				
Cardiovascular diseases	1 (5.5)	3 (7.3)	2 (8)	NS
Diabetes	2 (11.1)	5 (12.2)	4 (16)	NS
COPD	1 (5.5)	6 (14)	3 (12)	**<0.001**
Neurological diseases	1 (5.5)	3 (7.3)	1 (4)	NS
Psychiatric diseases	0 (0)	1 (2.4)	1 (4)	NS
Kidney diseases	1 (5.5)	2 (4.9)	2 (8)	NS
Malignancies ^3^	1 (5.5)	2 (4.9)	2 (8)	NS
WBCs (10^9^/L)	8346 [6155–9445]	8125 [6710–10,943]	7934 [6155–9934]	NS
Platelets (10^9^/L)	213 [171–319]	225 [199–278]	239 [182–285]	NS
eGFR (mL/min)	62.5 [49–72.5]	60.5 [43–78]	59.5 [41.5–77]	NS
CRP (mg/L)	12.6 [9.3–21.9]	14.5 [11.2–25.3]	11.4 [10.9–21.3]	NS
ALT (U/L)	31 [17–51]	30 [21–66]	25 [18–41]	NS
Ferritin (ng/mL)	630 [481–1040]	661 [552–1209]	600 [480–1124]	NS
D-dimer (ng/mL)	47 [18–190]	41 [26–168]	50 [27–189]	NS
NLR (ratio)	5.8 [2.5–9.3]	5.1 [1.8–8.7]	5.9 [3.1–7.4]	NS
HBV-DNA positivity (N) ^4^	4 (22.2)	0 (0)	1 (4)	**<0.001**
HBV-DNA (IU/mL)	189 [180–308]	-	66 ^5^	**<0.001**
Treatment with NAs (N)	3 (16.7)	0 (0)	0 (0)	**<0.001**
Corticosteroid treatment (N) ^6,7^	10 (55.5)	25 (60.9)	14 (56)	NS
Remdesivir treatment (N) ^6^	9 (50)	23 (56)	15 (60)	NS

Abbreviations: Alanine aminotransferase (ALT); hepatitis B core antibody (anti-HBc); hepatitis B surface antibody (anti-HBs); chronic obstructive pulmonary disease (COPD); C-reactive protein (CRP); estimated glomerular filtration rate (eGFR); hepatitis B surface antigen (HBsAg); hepatitis B virus (HBV); number (N); nucleoside analogues (NAs); neutrophil-to-lymphocyte ratio (NLR); nonsignificant (NS); white blood cells (WBCs); ^1^ these percentages refer to the total number of recruited subjects (N = 84); ^2^ at elastography (Fibroscan^®^); ^3^ all low-grade malignant tumors not in active treatment, with no current or previous hepatocarcinomas; ^4^ ≥20 IU/mL; ^5^ interquartile range not calculable for one single patient; ^6^ for COVID-19 treatment; ^7^ dexamethasone 6 mg/daily for 5–10 days.

**Table 2:** Outcomes observed in the study population. The percentages are referred to each column of this table. (**A**) All patients; (**B**) patients with HBV reactivation. Bold values denote statistical significance at the *p* < 0.05 level.

	HBsAg-Positive/ Anti-HBc-Positive (N = 18)	HBsAg-Negative, Anti-HBc-Positive		*p*
		Anti-HBs-Positive (N = 41)	Anti-HBs-Negative (N = 25)	
(**A**)				
Hospitalization time (days)	12.5 [9.5–14.0]	11.0 [8.5–12.5]	12.0 [9.5–14.5]	NS
ICU admission (N)	3 (16.7)	5 (12.2)	4 (16.0)	NS
CPAP/NIV support (N)	5 (27.8)	11 (26.8)	7 (28.0)	NS
Sepsis (N)	1 (5.5)	2 (4.8)	1 (4.0)	NS
Death (N)	1 (5.5)	1 (2.4)	2 (8.0)	**0.014** ^1^
(**B**)				
Only circulating detectable HBV-DNA (N)	2 ^2^ (11.0)	0 (0.0)	2 ^3^ (8.0)	NS
HBsAg seroreversion (N)	NA	0 (0.0)	0 (0.0)	NS
Hepatitis flare (N)	3 (16.7)	2 (4.8)	4 (16.0)	**0.003** ^1^
ALT peak (U/L)	667 [533–819]	204 ^4^	146 [124–196]	

Abbreviations: Alanine aminotransferase (ALT); hepatitis B core antibody (anti-HBc)*;* hepatitis B surface antibody (anti-HBs)*;* hepatitis B surface antigen (HBsAg)*;* hepatitis B virus (HBV); intensive care unit (ICU); continuous positive airway pressure (CPAP); number (N); not applicable (NA); noninvasive ventilation (NIV); nonsignificant (NS); ^1^ HBsAg-positive plus anti-HBs-negative vs. anti-HBs-positive; ^2^ baseline vs. detected HBV-DNA peaks, respectively: 190→890 IU/mL, 427→1286 IU/mL; ^3^ baseline vs. detected HBV-DNA peak, respectively: 66→776 IU/mL, negative→917 IU/mL; ^4^ interquartile range not calculable for two samples.

**Table 3:** Factors associated with HBV reactivation. (**A**) Univariate analysis; (**B**) multivariate analysis. Bold values denote statistical significance at the *p* < 0.05 level.

Factors	OR	95% CI	*p*
(**A**)			
Age (years)	1.224	0.916–14.783	0.893
Sex	1.789	0.915–11.313	0.776
Cardiovascular diseases	2.223	0.714–9.667	0.591
Diabetes	1.018	0.996–4.927	0.885
COPD	3.392	0.812–7.776	0.714
Neurological diseases	1.298	0.671–5.672	0.727
Psychiatric diseases	0.782	0.619–2.562	0.678
Kidney diseases	1.444	0.932–4.873	0.843
Malignancies	1.782	0.716–6.715	0.698
Hospitalization time	3.673	0.834–9.128	0.778
ICU admission	2.278	1.036–9.476	**0.012**
CPAP/NIV support	2.789	0.987–11.972	0.678
Sepsis	1.446	1.119–6.337	**0.014**
NLR	3.415	0.067–12.993	0.765
HBV-DNA positivity	1.014	0.772–12.443	0.746
Anti-HBs positivity	0.814	0.972–0.765	**0.012**
Corticosteroid treatment	4.017	2.018–19.338	**0.007**
Remdesivir treatment	1.114	0.986–6.718	0.783
(**B**)			
ICU admission	3.914	1.966–11.451	**0.008**
Sepsis	1.091	0.967–7.816	0.089
Anti-HBs positivity	0.804	0.713–0.987	**0.014**
Corticosteroid treatment	4.367	2.561–16.445	**0.002**

Abbreviations: Chronic obstructive pulmonary disease (COPD); continuous positive airway pressure (CPAP); hepatitis B virus (HBV); anti-hepatitis B surface antibodies (HBs); intensive care unit (ICU); noninvasive ventilation (NIV); neutrophil-to-lymphocyte ratio (NLR); odds ratio (OR).

## Data Availability

All data generated or analyzed during this study are included in this published article.

## References

[1] Wang C., Horby P.W., Hayden F.G., Gao G.F. (2020). A Novel Coronavirus Outbreak of Global Health Concern. Lancet.

[2] Guan W., Ni Z., Hu Y., Liang W., Ou C., He J., Liu L., Shan H., Lei C., Hui D.S.C. (2020). Clinical Characteristics of Coronavirus Disease 2019 in China. N. Engl. J. Med..

[3] Hu L., Chen S., Fu Y., Gao Z., Long H., Ren H.W., Zuo Y., Wang J., Li H., Xu Q.B. (2020). Risk Factors Associated with Clinical Outcomes in 323 Coronavirus Disease 2019 (COVID-19) Hospitalized Patients in Wuhan, China. Clin. Infect. Dis..

[4] Briel M., Spoorenberg S.M.C., Snijders D., Torres A., Fernandez-Serrano S., Meduri G.U., Gabarrús A., Blum C.A., Confalonieri M., Kasenda B. (2018). Corticosteroids in Patients Hospitalized with Community-Acquired Pneumonia: Systematic Review and Individual Patient Data Metaanalysis. Clin. Infect. Dis..

[5] Pirola C.J., Sookoian S. (2020). SARS-CoV-2 Virus and Liver Expression of Host Receptors: Putative Mechanisms of Liver Involvement in COVID-19. Liver Int..

[6] Ji D., Zhang D., Yang T., Mu J., Zhao P., Xu J., Li C., Cheng G., Wang Y., Chen Z. (2020). Effect of COVID-19 on Patients with Compensated Chronic Liver Diseases. Hepatol. Int..

[7] Boglione L., Rostagno R., Poletti F., Moglia R., Bianchi B., Esposito M., Biffi S., Borrè S. (2021). Letter: Liver Involvement and Mortality in COVID-19—The Role of Anti-Viral Therapy Should Be Considered. Aliment. Pharmacol. Ther..

[8] Boglione L., Corcione S., Shbaklo N., Rosso T., Lupia T., Pinna S.M., Scabini S., Ciccone G., De Benedetto I., Borrè S. (2022). Liver Involvement and Mortality in COVID-19: A Retrospective Analysis from the CORACLE Study Group. Infez. Med..

[9] Luo M., Ballester M.P., Soffientini U., Jalan R., Mehta G. (2022). SARS-CoV-2 Infection and Liver Involvement. Hepatol. Int..

[10] Yuen M.F., Chen D.S., Dusheiko G.M., Janssen H.L.A., Lau D.T.Y., Locarnini S.A., Peters M.G., Lai C.L. (2018). Hepatitis B Virus Infection. Nat. Rev. Dis. Primers.

[11] Alqahtani S.A., Buti M. (2021). COVID-19 and Hepatitis B Infection. Antivir. Ther..

[12] Guo Y., Zeng X., Li L., Wang L. (2023). The Impact of HBV Infection on Clinical Outcomes of COVID-19 Patients: A Systematic Review and Meta-Analysis. Epidemiol. Infect..

[13] Yip T.C.F., Wong V.W.S., Lui G.C.Y., Chow V.C.Y., Tse Y.K., Hui V.W.K., Liang L.Y., Chan H.L.Y., Hui D.S.C., Wong G.L.H. (2021). Current and Past Infections of HBV Do Not Increase Mortality in Patients With COVID-19. Hepatology.

[14] Wang J., Lu Z., Jin M., Wang Y., Tian K., Xiao J., Cai Y., Wang Y., Zhang X., Chen T. (2022). Clinical Characteristics and Risk Factors of COVID-19 Patients with Chronic Hepatitis B: A Multi-Center Retrospective Cohort Study. Front. Med..

[15] Spera A.M. (2022). Hepatitis B Virus Infection Reactivation in Patients under Immunosuppressive Therapies: Pathogenesis, Screening, Prevention and Treatment. World J. Virol..

[16] Foo H., Phan F., Bagatella M., Petrovski I., Nagendra V., Acharya P., Levy M., Prakoso E. (2023). Risk of Hepatitis B Reactivation Following Baricitinib or Tocilizumab for Treatment of COVID-19. Eur. J. Clin. Microbiol. Infect..

[17] Wong G.L.H., Wong V.W.S., Yuen B.W.Y., Tse Y.K., Yip T.C.F., Luk H.W.S., Lui G.C.Y., Chan H.L.Y. (2020). Risk of Hepatitis B Surface Antigen Seroreversion after Corticosteroid Treatment in Patients with Previous Hepatitis B Virus Exposure. J. Hepatol..

[18] Shi Y., Zheng M. (2020). Hepatitis B Virus Persistence and Reactivation. BMJ.

[19] Del Pozo J.L. (2021). Respiratory Co-and Superinfections in COVID-19. Rev. Esp. Quimioter..

[20] Traylen C.M., Patel H.R., Fondaw W., Mahatme S., Williams J.F., Walker L.R., Dyson O.F., Arce S., Akula S.M. (2011). Virus Reactivation: A Panoramic View in Human Infections. Future Virol..

[21] Liu Y., Li Y., Xu D., Zhang J., Peng Z. (2021). Severe COVID-19: Immunosuppression or Hyperinflammation?. Shock.

[22] Kim J.Y.H., Ragusa M., Tortosa F., Torres A., Gresh L., Méndez-Rico J.A., Alvarez-Moreno C.A., Lisboa T.C., Valderrama-Beltrán S.L., Aldighieri S. (2023). Viral Reactivations and Co-Infections in COVID-19 Patients: A Systematic Review. BMC Infect. Dis..

[23] Liu J., Wang T., Cai Q., Sun L., Huang D., Zhou G., He Q., Wang F.S., Liu L., Chen J. (2020). Longitudinal Changes of Liver Function and Hepatitis B Reactivation in COVID-19 Patients with Pre-Existing Chronic Hepatitis B Virus Infection. Hepatol. Res..

[24] Aldhaleei W.A., Alnuaimi A., Bhagavathula A.S. (2020). COVID-19 Induced Hepatitis B Virus Reactivation: A Novel Case From the United Arab Emirates. Cureus.

[25] Ciceri F., Castagna A., Rovere-Querini P., De Cobelli F., Ruggeri A., Galli L., Conte C., De Lorenzo R., Poli A., Ambrosio A. (2020). Early Predictors of Clinical Outcomes of COVID-19 Outbreak in Milan, Italy. Clin. Immunol..

[26] Maddaloni E., D’Onofrio L., Alessandri F., Mignogna C., Leto G., Pascarella G., Mezzaroma I., Lichtner M., Pozzilli P., Agrò F.E. (2020). Cardiometabolic Multimorbidity Is Associated with a Worse COVID-19 Prognosis than Individual Cardiometabolic Risk Factors: A Multicentre Retrospective Study (CoViDiab II). Cardiovasc. Diabetol..

[27] Figliozzi S., Masci P.G., Ahmadi N., Tondi L., Koutli E., Aimo A., Stamatelopoulos K., Dimopoulos M.A., Caforio A.L.P., Georgiopoulos G. (2020). Predictors of Adverse Prognosis in COVID-19: A Systematic Review and Meta-Analysis. Eur. J. Clin. Investig..

[28] Fadini G.P., Morieri M.L., Boscari F., Fioretto P., Maran A., Busetto L., Bonora B.M., Selmin E., Arcidiacono G., Pinelli S. (2020). Newly-Diagnosed Diabetes and Admission Hyperglycemia Predict COVID-19 Severity by Aggravating Respiratory Deterioration. Diabetes Res. Clin. Pract..

[29] Chang M.S., Nguyen M.H. (2017). Epidemiology of Hepatitis B and the Role of Vaccination. Best. Pract. Res. Clin. Gastroenterol..

[30] Liao F.L., Peng D.H., Chen W., Hu H.N., Tang P., Liu Y.Y., Luo Y., Yao T. (2021). Evaluation of Serum Hepatic Enzyme Activities in Different COVID-19 Phenotypes. J. Med. Virol..

[31] Du M., Yang S., Liu M., Liu J. (2022). COVID-19 and Liver Dysfunction: Epidemiology, Association and Potential Mechanisms. Clin. Res. Hepatol. Gastroenterol..

[32] He Y.F., Jiang Z.G., Wu N., Bian N., Ren J.L. (2022). Correlation between COVID-19 and Hepatitis B: A Systematic Review. World J. Gastroenterol..

[33] Wu J., Yu J., Shi X., Li W., Song S., Zhao L., Zhao X., Liu J., Wang D., Liu C. (2021). Epidemiological and Clinical Characteristics of 70 Cases of Coronavirus Disease and Concomitant Hepatitis B Virus Infection: A Multicentre Descriptive Study. J. Viral Hepat..

[34] Bekçibaşı M., Arslan E. (2021). Severe Acute Respiratory Syndrome Coronavirus 2 (SARS-CoV-2) /Hepatitis B Virus (HBV) Co-Infected Patients: A Case Series and Review of the Literature. Int. J. Clin. Pract..

[35] Yang S., Wang S., Du M., Liu M., Liu Y., He Y. (2022). Patients with COVID-19 and HBV Coinfection Are at Risk of Poor Prognosis. Infect. Dis. Ther..

[36] Kang S.H., Cho D.H., Choi J., Baik S.K., Gwon J.G., Kim M.Y. (2021). Association between Chronic Hepatitis B Infection and COVID-19 Outcomes: A Korean Nationwide Cohort Study. PLoS ONE.

[37] Xie Y., Choi T., Al-Aly Z. (2023). Risk of Death in Patients Hospitalized for COVID-19 vs Seasonal Influenza in Fall-Winter 2022-2023. JAMA.

[38] Hu F.H., Jia Y.J., Zhao D.Y., Fu X.L., Zhang W.Q., Tang W., Hu S.Q., Wu H., Ge M.W., Du W. (2023). Clinical Outcomes of the Severe Acute Respiratory Syndrome Coronavirus 2 Omicron and Delta Variant: Systematic Review and Meta-Analysis of 33 Studies Covering 6 037 144 Coronavirus Disease 2019–Positive Patients. Clin. Microbiol. Infect..

[39] Italian Ministry of Health Report on COVID-19 Vaccines. https://www.governo.it/it/cscovid19/report-vaccini/.

[40] Zhang J.J., Dong X., Liu G.H., Gao Y.D. (2023). Risk and Protective Factors for COVID-19 Morbidity, Severity, and Mortality. Clin. Rev. Allergy Immunol..

[41] Alemu J., Gumi B., Tsegaye A., Rahimeto Z., Fentahun D., Ibrahim F., Abubeker A., Gebremedhin A., Gelanew T., Howe R. (2024). Seroprevalence of SARS-CoV-2 and Hepatitis B Virus Coinfections among Ethiopians with Acute Leukemia. Cancers.

[42] Zou X., Fang M., Li S., Wu L., Gao B., Gao H., Ran X., Bian Y., Li R., Yu S. (2021). Characteristics of Liver Function in Patients with SARS-CoV-2 and Chronic HBV Coinfection. Clin. Gastroenterol. Hepatol..

[43] Elghannam M.T., Hassanien M.H., Ameen Y.A., ELattar G.M., ELRay A.A., Turky E.A., ELTalkawy M.D. (2022). COVID-19 and Liver Diseases. Egypt. Liver J..

[44] Choe J.W., Jung Y.K., Yim H.J., Seo G.H. (2022). Clinical Effect of Hepatitis B Virus on COVID-19 Infected Patients:A Nationwide Population-Based Study Using the Health Insurance Review & Assessment Service Database. J. Korean Med. Sci..

[45] Bellan M., Patti G., Hayden E., Azzolina D., Pirisi M., Acquaviva A., Aimaretti G., Aluffi Valletti P., Angilletta R., Arioli R. (2020). Fatality Rate and Predictors of Mortality in an Italian Cohort of Hospitalized COVID-19 Patients. Sci. Rep..

[46] Spinoni E.G., Mennuni M., Rognoni A., Grisafi L., Colombo C., Lio V., Renda G., Foglietta M., Petrilli I., D’Ardes D. (2021). Contribution of Atrial Fibrillation to In-Hospital Mortality in Patients with COVID-19. Circ. Arrhythm. Electrophysiol..

[47] Lin Y., Yuan J., Long Q., Hu J., Deng H., Zhao Z., Chen J., Lu M., Huang A. (2021). Patients with SARS-CoV-2 and HBV Co-Infection Are at Risk of Greater Liver Injury. Genes. Dis..

[48] Zhu J.H., Peltekian K.M. (2021). HBV Coinfection and In-Hospital Outcomes for COVID-19: A Systematic Review and Meta-Analysis. Can. Liver J..

[49] Liu R., Zhao L., Cheng X., Han H., Li C., Li D., Liu A., Gao G., Zhou F., Liu F. (2021). Clinical Characteristics of COVID-19 Patients with Hepatitis B Virus Infection—A Retrospective Study. Liver Int..

[50] Yu R., Tan S., Dan Y., Lu Y., Zhang J., Tan Z., He X., Xiang X., Zhou Y., Guo Y. (2021). Effect of SARS-CoV-2 Coinfection Was Not Apparent on the Dynamics of Chronic Hepatitis B Infection. Virology.

[51] Chen L., Huang S., Yang J., Cheng X., Shang Z., Lu H., Cheng J. (2020). Clinical Characteristics in Patients with SARS-CoV-2/HBV Co-Infection. J. Viral Hepat..

[52] Li Y., Li C., Wang J., Zhu C., Zhu L., Ji F., Liu L., Xu T., Zhang B., Xue L. (2020). A Case Series of COVID-19 Patients with Chronic Hepatitis B Virus Infection. J. Med. Virol..

[53] Xiang T.D., Zheng X. (2021). Interaction between Hepatitis B Virus and SARS-CoV-2 Infections. World J. Gastroenterol..

[54] Iavarone M., D’Ambrosio R., Soria A., Triolo M., Pugliese N., Del Poggio P., Perricone G., Massironi S., Spinetti A., Buscarini E. (2020). High Rates of 30-Day Mortality in Patients with Cirrhosis and COVID-19. J. Hepatol..

[55] Moon A.M., Webb G.J., Aloman C., Armstrong M.J., Cargill T., Dhanasekaran R., Genescà J., Gill U.S., James T.W., Jones P.D. (2020). High Mortality Rates for SARS-CoV-2 Infection in Patients with Pre-Existing Chronic Liver Disease and Cirrhosis: Preliminary Results from an International Registry. J. Hepatol..

[56] Sarin S.K., Choudhury A., Lau G.K., Zheng M.H., Ji D., Abd-Elsalam S., Hwang J., Qi X., Cua I.H., Suh J.I. (2020). Pre-Existing Liver Disease Is Associated with Poor Outcome in Patients with SARS-CoV-2 Infection; The APCOLIS Study (APASL COVID-19 Liver Injury Spectrum Study). Hepatol. Int..

[57] Kim D., Adeniji N., Latt N., Kumar S., Bloom P.P., Aby E.S., Perumalswami P., Roytman M., Li M., Vogel A.S. (2021). Predictors of Outcomes of COVID-19 in Patients with Chronic Liver Disease: US Multi-Center Study. Clin. Gastroenterol. Hepatol..

[58] Roche B., Samuel D. (2011). The Difficulties of Managing Severe Hepatitis B Virus Reactivation. Liver Int..

[59] Smalls D.J., Kiger R.E., Norris L.A.B., Bennett C.L., Love B.L. (2019). Hepatitis B Virus Reactivation: Risk Factors and Current Management Strategies. Pharmacotherapy.

[60] Sonneveld M.J., Murad S.D., van der Eijk A.A., de Man R.A. (2019). Fulminant Liver Failure Due to Hepatitis B Reactivation During Treatment with Tocilizumab. ACG Case Rep. J..

[61] Campbell C., Andersson M.I., Ansari M.A., Moswela O., Misbah S.A., Klenerman P., Matthews P.C. (2021). Risk of Reactivation of Hepatitis B Virus (HBV) and Tuberculosis (TB) and Complications of Hepatitis C Virus (HCV) Following Tocilizumab Therapy: A Systematic Review to Inform Risk Assessment in the COVID-19 Era. Front. Med..

[62] Cao B., Wang Y., Wen D., Liu W., Wang J., Fan G., Ruan L., Song B., Cai Y., Wei M. (2020). A Trial of Lopinavir–Ritonavir in Adults Hospitalized with Severe COVID-19. N. Engl. J. Med..

[63] Horby P., Lim W.S., Emberson J.R., Mafham M., Bell J.L., Linsell L., Staplin N., Brightling C., Ustianowski A., RECOVERY Collaborative Group (2021). Dexamethasone in Hospitalized Patients with Covid-19. N. Engl. J. Med..

[64] Chen F., Zong L., Li Y., Zhu H., Xu S., Xu J. (2024). Opportunity for Severe and Critical COVID-19 Pneumonia Treatment with Corticosteroids: A Retrospective Cohort Study. J. Thorac. Dis..

[65] Alonazi J., Alrasheed N., Aljabr S., Albaqami K., Alshallal K., Alsemairi S.A., AlBaqami F., Alnufaie N.F., Bin Talib F.A. (2024). Timing of Dexamethasone Initiation and Its Impact on the Outcome of COVID-19 Patients. Cureus.

[66] Reddy K.R., Beavers K.L., Hammond S.P., Lim J.K., Falck-Ytter Y.T. (2015). American Gastroenterological Association Institute Guideline on the Prevention and Treatment of Hepatitis B Virus Reactivation during Immunosuppressive Drug Therapy. Gastroenterology.

[67] Yip T.C.F., Gill M., Wong G.L.H., Liu K. (2022). Management of Hepatitis B Virus Reactivation Due to Treatment of COVID-19. Hepatol. Int..

[68] Librero Jiménez M., López Garrido M.Á., Fernández Cano M.C. (2022). Letter to the Editor: Reactivation of HBV Triggered by SARS-CoV-2 in a Patient with Cirrhosis. Hepatology.

[69] Mihai N., Olariu M.C., Ganea O.-A., Adamescu A.-I., Molagic V., Aramă Ș.S., Tilișcan C., Aramă V. (2024). Risk of Hepatitis B Virus Reactivation in COVID-19 Patients Receiving Immunosuppressive Treatment: A Prospective Study. J. Clin. Med..

[70] Gómez Camarero J., Badia Aranda E., Quiñones Castro R., Saiz Chumillas R.M., Alcoba Vega L., Díez Ruiz S., Gómez Manero N., Vinuesa Campo R., Jorquera Plaza F. (2022). Hepatitis B and C Screening in Hospitalized Patients with SARS-CoV-2 Infection. Gastroenterol. Hepatol..

